# Different Polar Metabolites and Protein Profiles between High- and Low-Quality Japanese *Ginjo* Sake

**DOI:** 10.1371/journal.pone.0150524

**Published:** 2016-03-03

**Authors:** Kei Takahashi, Hiromi Kohno

**Affiliations:** National Research Institute of Brewing, 3-7-1 Kagamiyama, Higashi-hiroshima, Hiroshima, 739–0046, Japan; The University of Tokyo, JAPAN

## Abstract

Japanese *ginjo* sake is a premium refined sake characterized by a pleasant fruity apple-like flavor and a sophisticated taste. Because of technical difficulties inherent in brewing *ginjo* sake, off-flavors sometimes occur. However, the metabolites responsible for off-flavors as well as those present or absent in higher quality *ginjo* sake remain uncertain. Here, the relationship between 202 polar chemical compounds in sake identified using capillary electrophoresis coupled with time-of-flight mass spectrometry and its organoleptic properties, such as quality and off-flavor, was examined. First, we found that some off-flavored sakes contained higher total amounts of metabolites than other sake samples. The results also identified that levels of 2-oxoglutaric acid and fumaric acid, metabolites in the tricarboxylic acid cycle, were highly but oppositely correlated with *ginjo* sake quality. Similarly, pyridoxine and pyridoxamine, co-enzymes for amino transferase, were also highly but oppositely correlated with *ginjo* sake quality. Additionally, pyruvic acid levels were associated with good quality as well. Compounds involved in the methionine salvage cycle, oxidative glutathione derivatives, and amino acid catabolites were correlated with low quality. Among off-flavors, an inharmonious bitter taste appeared attributable to polyamines. Furthermore, protein analysis displayed that a diversity of protein components and yeast protein (triosephosphate isomerase, TPI) leakage was linked to the overall metabolite intensity in *ginjo* sake. This research provides insight into the relationship between sake components and organoleptic properties.

## Introduction

Japanese sake, a traditional fermented alcoholic beverage, is produced from polished rice (*Oryza sativa* Japonica sp.), rice mold (*Aspergillus oryzae*), and sake yeast (*Saccharomyces cerevisiae*) by simultaneous saccharification and alcohol fermentation [1, 2]. Rice mold produces and secretes a large amount of starch hydrolyzing enzymes and protein breakdown enzymes. In addition, rice mold produces lipids, amino acids, vitamins, and secondary metabolites. Sake yeast, in turn, produces ethanol and several types of higher alcohols, acetic ester, ethyl esters, and many types of organic acids. Sake thus contains a variety of volatile and non-volatile metabolites derived from rice and microorganisms, which often differ depending on the brewing process and conditions, techniques employed, pasteurization process, and the storage conditions [3–8], similar to that which occurs during winemaking [9–11] and beer brewing [12–14] processes. Among Japanese sake, *ginjo* sake, a premium sake (refined rice wine), has become popular even abroad. In the *ginjo* brewing process, sake mash is fermented for a long period (approximately 30–40 days) under low temperature to carefully control yeast and enzymatic activity, and to not emit fruity ester compounds into the air. Owing to the technical difficulties of handling *ginjo* sake mash, which arise in part because of the higher ethyl ester-producing cerulenin-resistant sake yeast generally used in modern *ginjo* sake brewing, undesirable off-flavors can be accidentally albeit rarely generated, leading to a degraded *ginjo* sake quality.

Many terms have been used to describe the characteristic tastes and flavors of sake, according to a Japanese government report [15]. With regard to pleasant flavor, ethyl hexanoate (ethyl caproate) [16] and Isoamyl acetate [17] produce fruity apple and banana like flavors, respectively. However, the molecular mechanisms underlying the generation of these compounds are considerably different. The typical ethyl esters in *ginjo* sake, ethyl hexanoate, is preferably produced at a lower temperature in sake mash than is isoamyl acetate; hence the ascertainment of ethyl hexanoate is thought to be one of the “*merkmale*” of higher quality of *ginjo* sake. With regard to pleasant taste, aftertaste, smoothness, body, and sweetness are important for *ginjo* sake. Conversely, with regard to unpleasant flavor, fatty acid [6, 7, 18], roast, deterioration “*hineka*” [19–21], deterioration arising from non-pasteurization “raw-*hineka*” [21] (similar to “*mureka*” [22]), yeast-debris, sulfur compound-like [23], diacetyl [24], and pungent (similar to 4-vinylguaiacol-like and smoke-like odors) odors occasionally arise during sake brewing. Unpleasant tastes, such as inharmonious bitter taste and inharmonious body also degrade *ginjo* sake quality. These organoleptic properties arise from the concentration or combination of chemical compounds in the sake. Previously, numerous studies have identified the compounds that confer such organoleptic properties in sake [3, 5, 6, 19–21, 23–25]. However, a comprehensive understanding of the different compounds and the resulting organoleptic properties such as quality and off-flavor is yet to be determined.

As a rudimentary challenge, a wide range of chemical compounds in sake have been detected [8, 25, 26], which has built a fundamental knowledge of the components in sake and their relationships to its sensory properties. Recently, the development of metabolite analytical instruments and data mining techniques have permitted a comprehensive analysis of the components in sake, and the associated correlation analyses of a variety of metabolites in sake with its sensory evaluation have just begun to be explored by several independent groups [3–6]. Using capillary electrophoresis time-of-flight mass spectrometry (CE–TOFMS), Sugimoto et al. found that the levels of amino acids and organic acids were positively correlated to the sourness of sake [3]. They also found that the pasteurization process considerably changes the metabolites in sake, and that the amino acids were reduced over the course of time during the storage period [4]. Mimura et al. used gas chromatography coupled with MS (GC–MS) to follow compound derivatization, and demonstrated that their strategy yielded results consistent with previous findings for an analysis based on orthogonal projections to latent structures regression analysis and variable importance on projection [5]. In our previous studies [6, 21], we used two dimensional GC coupled with TOFMS (GC × GC–TOFMS) to elucidate the correlations of volatile metabolites with organoleptic properties in *ginjo* sake by means of a check-all-that-apply (CATA) method and a quantitative descriptive analysis (QDA). These nascent metabolomics studies of sake have facilitated the examination of the methodologies used to investigate the associations between the components and organoleptic properties of sake. In addition, some relevant relationships between the components and organoleptic properties of sake have also been unveiled. However, the important enigma regarding the sake metabolites that are involved in the generation of off-flavors as well those present or absent in higher quality *ginjo* sake remains to be deciphered.

In addition to metabolomics, targeted protein and proteomics analyses of wine [27, 28], beer [29–35], and their corresponding ingredients such as grape [36–39], barley [29–32, 34], wheat [33], and yeast [31, 40–43], have progressed recently as well. Metabolites contained in fermented alcoholic beverages are thought to be highly associated with the protein entities that are leaked to the product from corresponding ingredients and microorganisms. Therefore, an approach that integrates the results of protein analysis into those of the metabolome is particularly effective for sake metabolite research.

In this study, we performed a correlation analysis of the polar metabolites in *ginjo* sake as analyzed using CE–TOFMS with the organoleptic properties (quality and off-flavor) of *ginjo* sake. Compounds in the central metabolic pathway, glutathione pathway, vitamin B_6_, and amino acid catabolites were associated with *ginjo* sake quality, whereas medium-chain fatty acids and compounds in the methionine salvage pathway were correlated with an unpleasant fatty acid odor. Polyamines were highly correlated with an unpleasant inharmonious bitter taste. Because some low quality *ginjo* sake contained higher amount of metabolites, protein analysis was performed to clarify whether the protein profile was associated with the metabolite level.

## Materials and Methods

### Materials

Thirteen premium Japanese sake samples brewed from *Yamadanishiki* rice cultivar were selected from Annual National New Sake Awards entries (National Research Institute of Brewing (NRIB) competition, 2011), as described previously [6]. For glucose quantification, a Wako glucose kit (Wako Pure Chemical Industries Ltd., Osaka, Japan) was used. For pyruvic acid quantification, F-kit pyruvate 8000J was purchased from J.K. International Inc. (Tokyo, Japan). Coomassie brilliant blue (CBB) R-250 was purchased from Nacalai Tesque Inc. (Kyoto, Japan). Other chemicals were purchased from Wako Pure Chemical Industries Ltd.

### Sensory Evaluations

Sensory evaluations were performed by 15 well-trained panelists as described in a Japanese government report [44]. The “taste quality” and “flavor quality” were evaluated using a 5-point scale.

Identification of specific organoleptic properties (fatty acid odor, inharmonious bitter taste, yeast debris-like odor, sulfur-like odor, and “*koji*” (rice mold) odor) was determined based on an assessment of their presence or absence in a sample using a CATA survey [45–47]. The number of notations of the presence of each factor was used as an index of intensity (Table 1).

**Table 1:** The quality score and the number of counts of *ginjo* sake by 15 sensory evaluation panelists.

Sample name	Flavor quality ^(a)^	Taste quality ^(a)^	Ethyl caproate	*Hineka* odor	Raw-*hineka* odor	Yeast debris-like odor	Sulfur-like odor	Fatty acid odor	Pleasant bitter taste	Pleasant astringent taste	Inharmonious bitter taste	Reference
**A1**	30	34	7	0	0	0	0	0	2	0	0	21
**A3**	40	38	5	0	1	0	0	0	4	4	1	21
**A4**	35	36	3	0	0	0	0	0	0	3	1	This study
**A5**	37	34	8	0	0	0	0	0	2	2	1	This study
**A9**	40	41	1	0	0	0	0	1	4	2	1	21
**A10**	34	33	3	0	0	0	0	3	3	5	2	21
**A11**	31	31	8	0	0	0	0	2	4	0	0	21
**B4**	43	46	1	0	2	1	1	1	2	4	5	21
**B5**	56	49	0	0	1	1	0	0	1	2	5	21
**B6**	71	63	1	3	1	3	4	0	2	0	6	21
**C2**	63	57	2	0	4	4	2	5	0	1	0	21
**C3**	73	63	2	6	3	3	4	5	0	0	0	21
**C5**	52	47	5	0	1	2	0	5	1	1	1	21

^(a)^ Low score means high quality

### Sample Collection and Preparation for CE–TOFMS Metabolomics Analysis

Japanese *ginjo* sake (80 μL) was mixed with 20 μL of 1,000μM internal standard solution 1 (Solution ID: H3304-1002, Human Metabolome Technologies (HMT), Tsuruoka, Japan). Then, the mixture was centrifugally filtered through a Millipore 5-kDa cutoff filter to remove proteins for 60 min at 9,100 × *g* and 4°C. The flow-through fraction was diluted to 2 and 5 fold by water for cation and anion mode analysis, respectively.

### CE–TOFMS Metabolomics Analysis

CE–TOFMS was carried out using an Agilent CE Capillary Electrophoresis System equipped with an Agilent 6210 TOFMS, Agilent 1100 isocratic HPLC pump, Agilent G1603A CE-MS adapter kit, and Agilent G1607A CE–ESI–MS sprayer kit (Agilent Technologies, Waldbronn, Germany). The system was controlled by Agilent G2201AA ChemStation software version B.03.01 for CE (Agilent Technologies).

Cationic metabolites were analyzed with a fused silica capillary (50μm i.d. × 80cm total length), with commercial cation electrophoresis buffer (Solution ID: H3301-1001, HMT) as the electrolyte. The sample was injected at a pressure of 50 mbar for 10s (approximately 10nL). The applied voltage was set at 27kV. Electrospray ionization–MS (ESI–MS) was conducted in the positive ion mode, and the capillary voltage was set at 4,000 V. The spectrometer was scanned from *m/z* 50 to 1,000.

Anionic metabolites were analyzed with a fused silica capillary (50μm i.d. × 80 cm total length), with commercial anion electrophoresis buffer (Solution ID: H3302-1021, HMT) as the electrolyte. The sample was injected at a pressure of 50 mbar for 25s (approximately 25nL). The applied voltage was set at 30kV. ESI–MS was conducted in the negative ion mode, and the capillary voltage was set at 3,500 V. The spectrometer was scanned from *m/z* 50 to 1,000.

Two independent CE–TOFMS analyses were examined. For quantitative analysis of the major compounds of the central metabolites pathway, each compound was calibrated by one-point using a 100 μM standard.

### Data Analysis

Raw data obtained by CE–TOFMS were processed with MasterHands [48]. Signal peaks corresponding to isotopomers, adduct ions, and other product ions of known metabolites were excluded, and all signal peaks potentially corresponding to authentic compounds were extracted, and then their migration time (MT) was normalized using those of the internal standards. Thereafter, the alignment of peaks was performed according to the *m/z* values and normalized MT values. Finally, peak areas were normalized against those of the internal standards, methionine sulfone and cyclosporin A for cations and anions, respectively. Annotation tables were produced from the CE–ESI–TOFMS measurements of standard compounds, and were aligned with the datasets according to similar *m/z* values and normalized MT values. The compounds that had statistically invalid area deviations by ANOVA (F value < 2, n = 13, 2) were excluded from further analysis. Principal component analysis (PCA) and correlation analyses were conducted using JMP version 10.0.2 (SAS Institute Inc., Cary, NC, USA).

### Polyclonal Antibodies

An anti-*S*. *cerevisiae* triosephosphate isomerase 1 (TPI1) antibody produced in a rabbit (LS-C147663) was purchased from LifeSpan BioSciences, Inc. (Seattle, WA, USA).

An anti-glucoamylase antibody was generated against the keyhole limpet hemocyanin (KLH)-conjugated synthetic peptides as cysteine + 114−127 amino acid sequence of *A*. *oryzae* glucoamylase GlaB (C+NEQAVSNPSGGLSD) from accession number BAA25205 [49]. The GlaB epitope sequence has some similarity with the 114–127 amino acid sequence of GlaA (accession number, P36914) [50]. An anti-α-amylase antibody was generated against the KLH-conjugated synthetic peptides as cysteine + 354−367 amino acid sequence of *A*. *oryzae* α-amylase, AmyA, AmyB, and AmyC, (C+QHYAGGNDPANREA) from accession number CAA31220 [51]. An anti-PepA antibody was generated against the KLH-conjugated synthetic peptides as cysteine + 236−254 amino acid sequence of *A*. *oryzae* acid protease, PepA (C+KYHAPGSYDFGFIDKSKFT) from accession number BAA02994 [52]. All three antibodies were raised in rabbits. After sampling each serum, IgG were purified by affinity chromatography using corresponding synthetic peptide conjugated columns. The generated antibodies were subdivided and kept at −80°C until thawed.

### Acetone Protein Precipitation

To cleave the *N*-linked glycosylation of proteins in sake, 1 mL sake was mixed with 1 μL PNGase F (500 U: New England Biolabs Japan, Tokyo, Japan) and incubated for 2 h at 0°C. PNGase F treated or untreated sake (1 mL) was mixed with an equal amount of acetone and kept at −80°C overnight. Following removal of the supernatant after centrifugation at 17,000 × *g* for 15 min, the precipitate was suspended in 40 μL solubilizing buffer containing 4 M urea, 6% SDS, 50 mM Tris-HCl (pH 6.8), 20% glycerol, and 2% DTT. After addition of 10 μL 5× Ling’s solubilizing buffer (50 mM Tris-HCl (pH 8.0), 5 mM EDTA, 10% SDS, 200 mM DTT, and 50% sucrose), protein was denatured at 50°C for 15 min. Then, 50 μL 2× urea buffer (10 M urea, 40 mM DTT, 10 mM Tris-HCl (pH 8.0), 1 mM EDTA, 2% SDS, and 10% sucrose) was added to the sample.

### SDS-PAGE and Immunoblot Analysis

SDS-PAGE was performed in 15% polyacrylamide gel (ATTO, Tokyo, Japan) with a size marker (Bio-Rad Laboratories, Hercules, MA, USA). After separation, proteins on the gel were stained with 0.1% CBB R-250. Then, the gel was destained at least for 12 h, and directly scanned using an EPSON GT 900 (Nagano, Japan).

For immunoblot analysis, following SDS-PAGE in 10% polyacrylamide gel (ATTO), proteins were transferred to a 0.22 μm pore PVDF membrane (Whatman) at 12 V, 60 mA, for 30 min. The membrane was soaked in blocking buffer (Nacalai Tesque, Inc.). After incubation with the respective primary antibodies (1/2000–1/4000 dilution), the membrane was washed three times with PBS containing Tween 20. The membrane was incubated with secondary antibodies conjugated with HRP (1/8000 dilution), followed by three washes with PBS containing Tween 20. Protein was detected by chemical luminescence (ECL prime, GE Healthcare Japan, Tokyo, Japan) using a ChemiDoc^™^ (Bio-Rad).

### Protein Identification in *Ginjo* Sake by MALDI–TOF/TOF

The gel pieces for spots of interest were obtained from 15% SDS-PAGE gels (ATTO) and digested in-gel using trypsin (Proteomics Sequencing Grade; Sigma, St. Louis, MO, USA) following destaining of CBB. Protein identification in the precipitates of *ginjo* sake was performed by using an Ultraflex TOF/TOF matrix-assisted laser desorption-ionization TOFMS system (MALDI–TOFMS: Bruker Daltonics, Billerica, MA, USA). Parameters for the analysis were as follows; mode of operation, reflection positive; accelerating voltage, 25 kV; acquisition mass range, 800–3,500 Da; MALDI matrix, α-cyano-4-hydroxycinnamic acid; calibration, external. The peptide fragment ion data obtained by MALDI–TOF/TOF were searched against the NCBI database of the origin to *S*. *cerevisiae*, *A*. *oryzae*, and *O*. *sativa* using Mascot software (Matrix Science, London, UK).

## Results

### Metabolomics of *Ginjo* Sake Using CE–TOFMS

**Fig 1:**
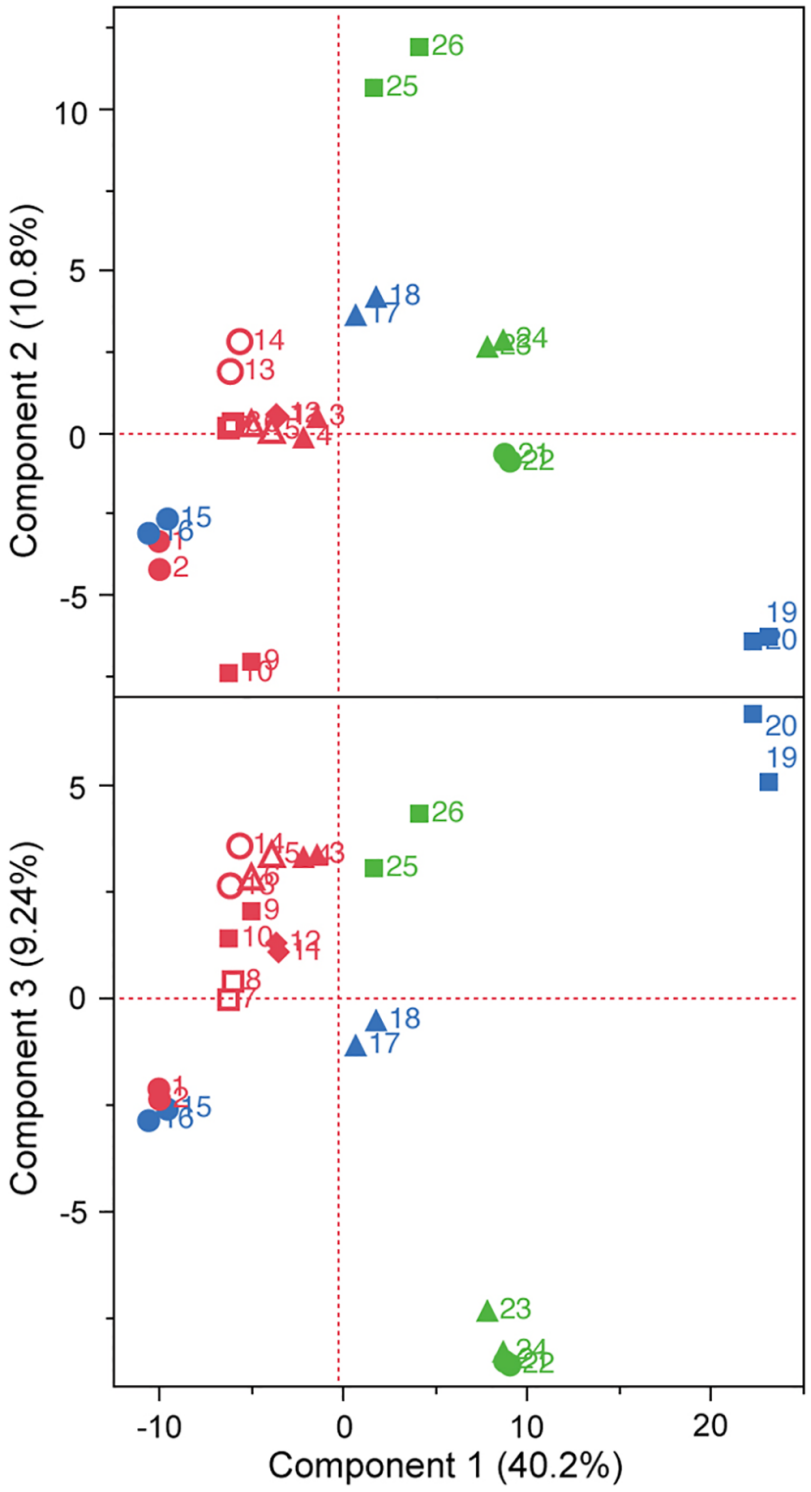
Principal component analysis (PCA) of Japanese sake. After selecting 202 compounds using Fisher ratio criteria, PCA was performed for 13 sake samples in duplicate. Same symbols indicate the same sake samples. Highly ranked sake (n = 7) are shown in red, sake with fatty acid odor (n = 3) are shown in green, and inharmonious bitter tasting sake (n = 3) are shown in blue.

**Fig 2:**
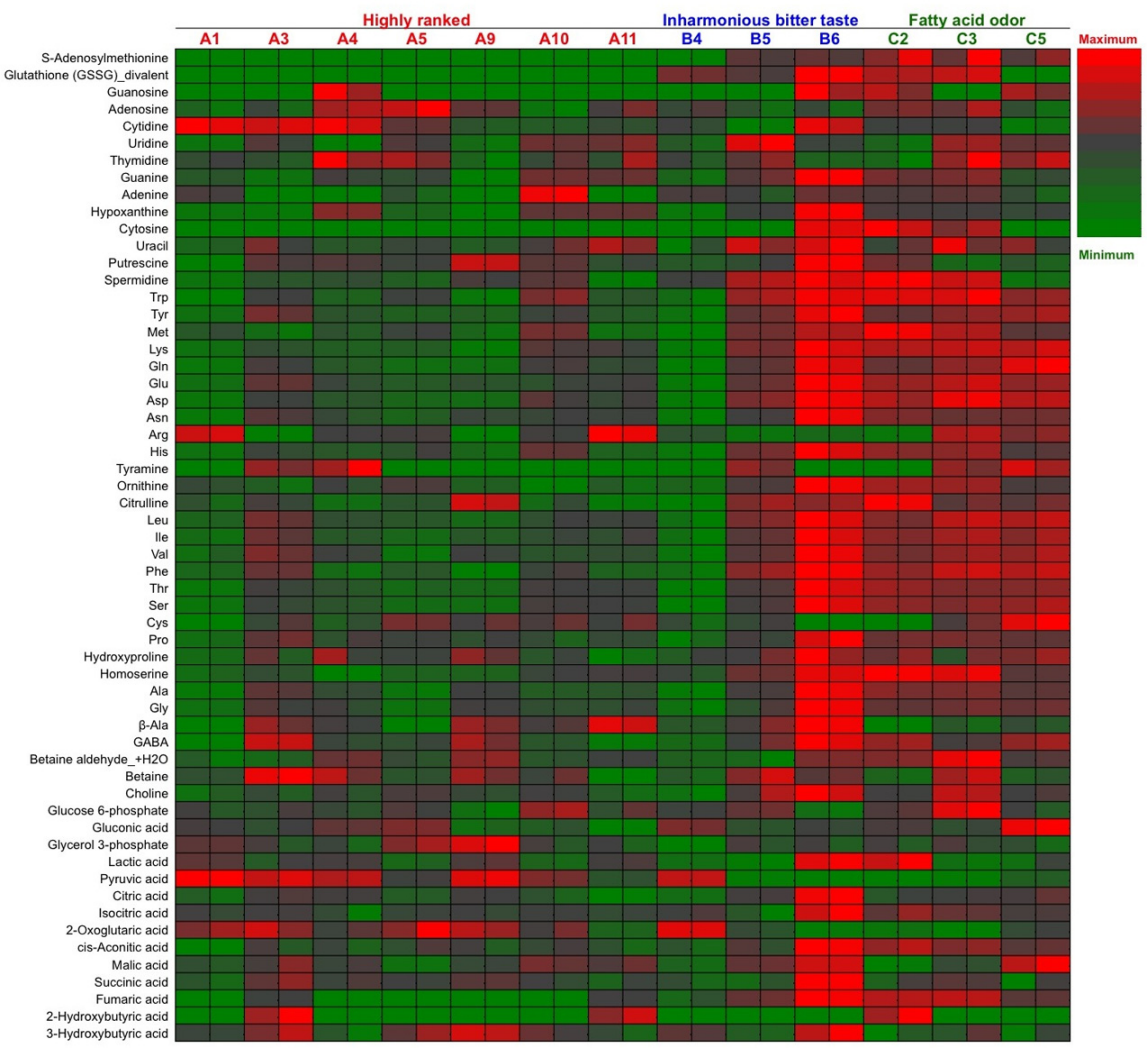
Quantified heatmap for the major metabolites of 13 sake samples. Heatmap showing the quantified metabolic profiles of 13 sake samples (7 of highly ranked sake, 3 of inharmonious bitter tasting sake, and 3 of sake with fatty acid odors) analyzed in duplicate. Maximum to minimum level is represented by a red-gray-green color scheme.

**Fig 3:**
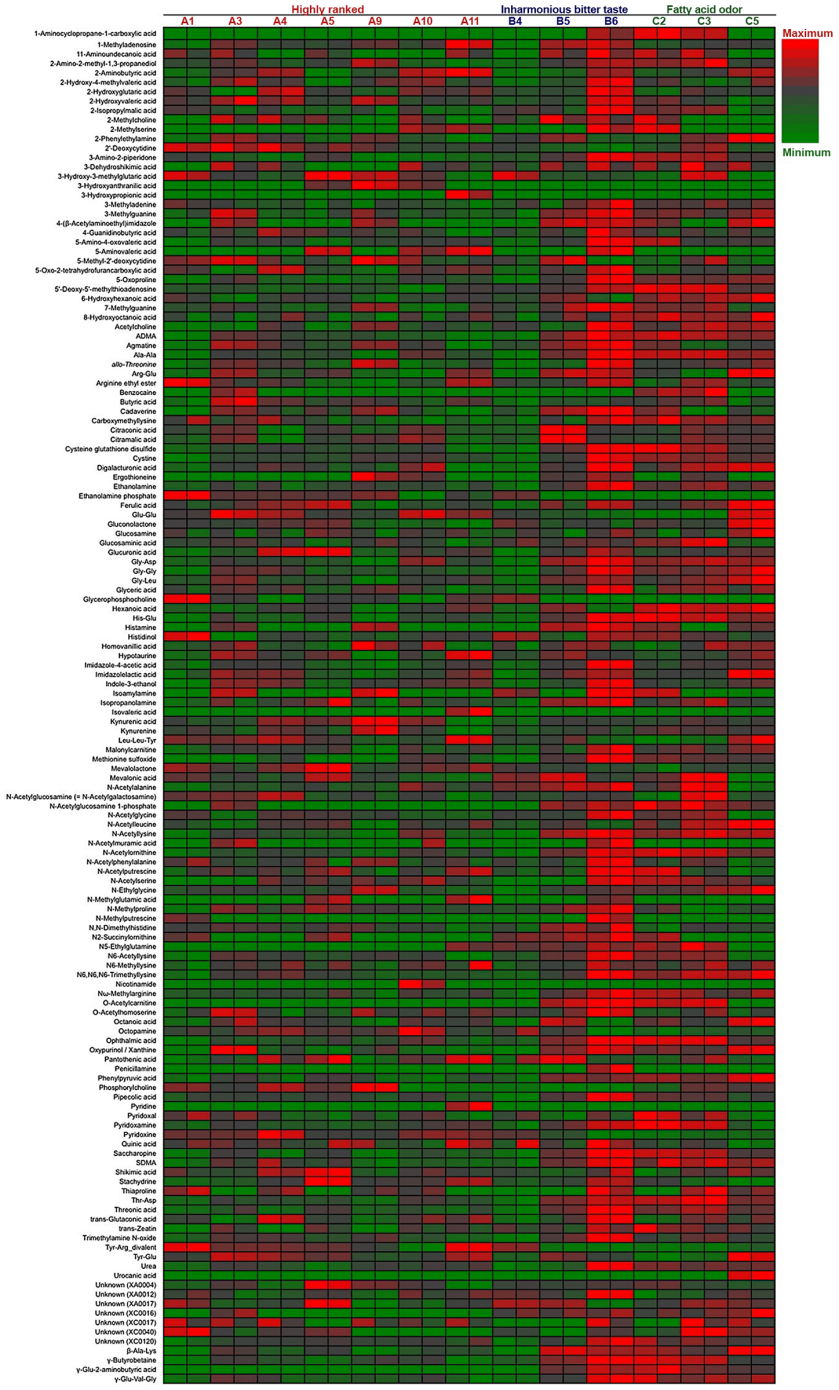
Relative heatmap for the metabolites of 13 sake samples. Heatmap showing the metabolic profiles of 13 sake samples (7 of highly ranked sake, 3 of inharmonious bitter tasting sake, and 3 of sake with fatty acid odors) analyzed in duplicate. Maximum to minimum level is represented by a red-gray-green color scheme. The values of *m/z* of the unknown compounds XA0004, XA0012, XA0017 are 144.0292, 166.0154, and 186.1124, respectively, by the anion detection mode. The values of *m/z* of the unknown compounds XC0016, XC0017, XC0040, and XC0120 are 129.0646, 130.0977, 174.0861, and 298.0518, respectively, by the cation detection mode.

The polar metabolites in 13 Japanese *ginjo* sake (refined rice wine) samples were analyzed by CE–TOFMS. From this, 156 cationic compounds, 62 anionic compounds, and up to 218 compounds in total were detected and annotated. Following exclusion of a small number of compounds by ANOVA calculations of Fisher ratios (F > 2, n = 13, 2), 202 compounds (148 cationic and 54 anionic, including unknown compounds) were further analysis by PCA. As shown in Fig 1, PCA showed that all 7 higher ranked sake were located to the left on the x-axis according to component 1, and 5 of 6 off-flavored low quality sake were located to the right, some of which were excessive. The metabolite containing patterns of sake samples A1 and B4 were similar. The heatmaps of polar metabolites are depicted in Figs 2 and 3, according to quantitative and relative values, respectively. Notably, the metabolite patterns of higher ranked sake were considerably different among the samples, suggesting diversity of taste and flavor among higher ranked sake. Apparently, some off-flavored sake (B6, C2, C3, C5), particularly sample B6, contained higher amounts of metabolites in total compared to other sake samples. This suggests that some off-flavored sake, which has relatively higher yeast debris-like odor and sulfur-like odor indices (Table 1), is apt to contain a large number of metabolites. The differences of metabolite containing patterns as shown in the heatmaps were corroborated by PCA in Fig 1. In addition, the similarity of metabolite patterns between A1 and B4 seen by PCA was also corroborated by the heatmaps in Figs 2 and 3.

### Correlations of Metabolites with *Ginjo* Sake Quality

Next, a correlation analysis of polar metabolites with sake quality using intensity by QDA as indicated in Table 1 was performed. The correlation coefficient of the taste and the flavor quality as well as the corresponding annotated compounds were listed (Table 2). Because the correlation coefficient between the taste and flavor quality validated by QDA in the *ginjo* sake used in this study was > 0.95 (Table 1), the corresponding compounds were quite similar between the two qualities (Table 2). The number of compounds with a negative value for the correlation coefficient (< −0.5) was lower than the number of compounds with a positive value (> 0.7), suggesting that many polar compounds can be considered as candidates for determining low quality *ginjo* sake, but that few polar compounds can be considered as determination factors for higher quality sake. The compounds that were abundant in high quality sake were as follows: pyridoxine, Glu-Glu, 2-oxoglutaric acid, pyruvic acid, octopamine, Leu-Leu-Tyr, Tyr-Arg, mevalolactone, 2′-deoxycytidine, ethanolamine phosphate, and Tyr-Glu (Table 2). Conversely, the representative compounds that were abundant in low quality sake were as follows: fumaric acid, gamma-butyrobetaine, ophthalmic acid, Thr-Asp, homoserine, saccharopine, Trp, His-Glu, 5′-deoxy-5′-methylthioadenosine, oxiglutathione (GSSG), Glu, cysteine glutathione disulfide, ornithine, pyridoxamine, and many amino acids except for arginine (Table 2).

**Table 2:** Chemical compounds whose levels were positively or negatively correlated with either taste or flavor quality.

Taste quality ^(a)^		Flavor quality ^(a)^	
Chemical compounds	*R*	Chemical compounds	*R*
Fumaric acid	0.913	Fumaric acid	0.928
γ-Butyrobetaine	0.904	Thr-Asp	0.928
Ophthalmic acid	0.896	Ophthalmic acid	0.925
Homoserine	0.895	γ-Butyrobetaine	0.914
Glutathione (GSSG)_divalent	0.893	*O*-Acetylcarnitine	0.914
*O*-Acetylcarnitine	0.891	Homoserine	0.907
Saccharopine	0.889	Saccharopine	0.902
Thr-Asp	0.877	Trp	0.900
5'-Deoxy-5'-methylthioadenosine	0.872	Ala-Ala	0.894
3-Amino-2-piperidone	0.864	*N*-Acetylornithine	0.892
Spermidine	0.862	His-Glu	0.888
*N*-Acetylornithine	0.846	Glu	0.876
His-Glu	0.846	5'-Deoxy-5'-methylthioadenosine	0.875
Cysteine glutathione disulfide	0.842	Threonic acid	0.874
Ornithine	0.836	3-Amino-2-piperidone	0.873
Trp	0.836	Spermidine	0.873
Ala-Ala	0.833	Asymmetry dimethyl arginine	0.873
Threonic acid	0.819	Glutathione (GSSG)_divalent	0.866
Glu	0.818	Phe	0.864
cis-Aconitic acid	0.817	Ile	0.845
Asymmetry dimethyl arginine	0.813	Cysteine glutathione disulfide	0.843
*N*-Acetylglucosamine 1-phosphate	0.801	His	0.841
*N*^6^-Acetyllysine	0.798	Ornithine	0.838
Phe	0.794	cis-Aconitic acid	0.836
Ile	0.791	*N*-Acetyllysine	0.835
Pyridoxamine	0.790	*N*-Acetylglucosamine 1-phosphate	0.832
Met	0.789	Leu	0.830
Ala	0.787	Met	0.812
Cytosine	0.785	Asp	0.812
His	0.781	Pyridoxamine	0.811
Val	0.775	Val	0.809
1-Aminocyclopropane-1-carboxylic acid	0.770	Ala	0.808
γ-Glu-Val-Gly	0.762	γ-Glu-Val-Gly	0.807
γ-Glu-2-aminobutyric acid	0.760	*N*^6^-Acetyllysine	0.806
Leu	0.757	Cystine	0.796
Cystine	0.756	Thr	0.794
N-Acetyllysine	0.752	Ethanolamine	0.786
*N*^5^-Ethylglutamine	0.749	Tyr	0.786
Acetylcholine	0.748	*N*^6^,*N*^6^,*N*^6^-Trimethyllysine	0.784
Ethanolamine	0.747	Symmetry dimethyl arginine	0.776
2-Isopropylmalic acid	0.747	Cytosine	0.773
7-Methylguanine	0.739	Gly-Asp	0.771
2-Amino-2-methyl-1,3-propanediol	0.735	1-Aminocyclopropane-1-carboxylic acid	0.767
Thr	0.733	Choline	0.759
Tyr	0.726	γ-Glu-2-aminobutyric acid	0.759
Asp	0.723	Lys	0.758
Asn	0.721	Asn	0.753
*N*^6^,*N*^6^,*N*^6^-Trimethyllysine	0.715	*N*^5^-Ethylglutamine	0.750
Glucosaminic acid	0.715	5-Oxoproline	0.748
Gly	0.708	Pro	0.748
Symmetry dimethyl arginine	0.707	Ser	0.747
5-Oxoproline	0.704	2-Amino-2-methyl-1,3-propanediol	0.740
Pro	0.703	Gly	0.738
		Acetylcholine	0.732
		7-Methylguanine	0.724
		2-Isopropylmalic acid	0.717
		*N*ω-Methylarginine	0.710
Shikimic acid	-0.206		
3-Hydroxyanthranilic acid	-0.207		
Glycerol 3-phosphate	-0.212		
Phosphorylcholine	-0.226		
Arg	-0.244		
Hypotaurine	-0.266		
5-Methyl-2'-deoxycytidine	-0.276		
Nicotinamide	-0.277		
Glycerophosphocholine	-0.287		
N-Methylglutamic acid	-0.290		
3-Hydroxypropionic acid	-0.327		
Isovaleric acid	-0.327		
Pyridine	-0.327	Glycerophosphocholine	-0.392
Ethanolamine phosphate	-0.409	Tyr-Glu	-0.392
Tyr-Glu	-0.475	Ethanolamine phosphate	-0.510
2'-Deoxycytidine	-0.527	2'-Deoxycytidine	-0.546
Tyr-Arg_divalent	-0.619	Mevalolactone	-0.632
Mevalolactone	-0.670	Leu-Leu-Tyr	-0.643
Leu-Leu-Tyr	-0.682	Tyr-Arg_divalent	-0.675
Pyruvic acid	-0.712	Octopamine	-0.728
Octopamine	-0.724	Glu-Glu	-0.753
2-Oxoglutaric acid	-0.733	2-Oxoglutaric acid	-0.772
Glu-Glu	-0.783	Pyruvic acid	-0.801
Pyridoxine	-0.861	Pyridoxine	-0.879

^(a)^ Negative value of correlation coefficient shows high quality.

### Metabolite Patterns in *Ginjo* Sake in the Central Metabolic Pathway

**Fig 4:**
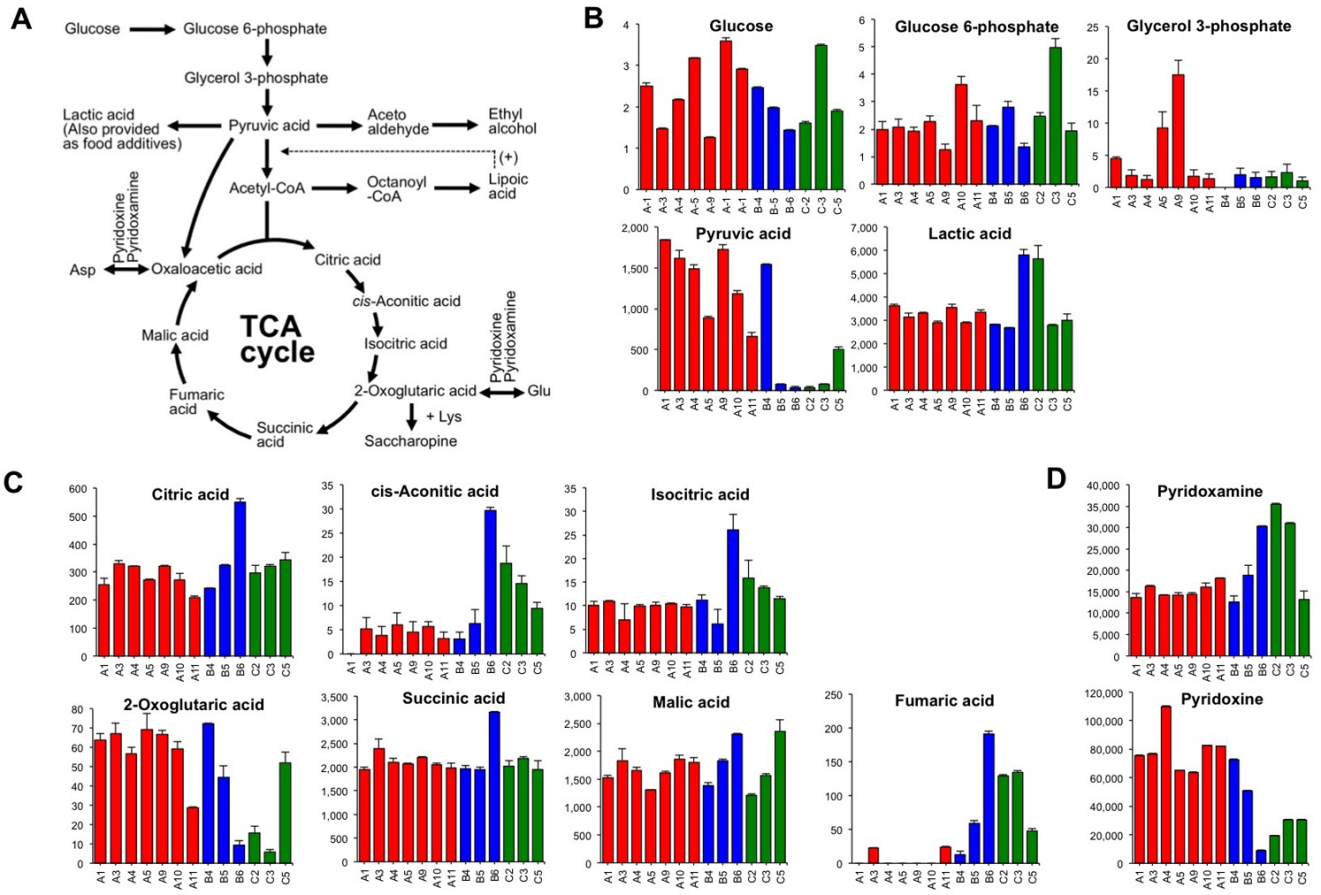
Patterns of identified metabolites from the central metabolic pathway of the sake yeast, *Saccharomyces cerevisiae*. A) A typical schematic for the central metabolic pathway. In the sake brewing process, lactic acid can be also added as a food additive at an early stage of the yeast starter making process. Alpha-lipoic acid can facilitate acetyl-CoA production with vitamin B_1_. B) The concentrations of the indicated chemical compounds involved in the glycolytic pathway in sake. Highly ranked sake, inharmonious bitter tasting sake, and sake with fatty acid odor are shown in red, blue, and green, respectively. Glucose is shown as a percentage (n = 4). Glucose 6-phosphate, glycerol 3-phosphate, pyruvic acid, and lactic acid are shown as μmol/L (n = 2). C) The concentrations of the indicated chemical compounds involved in the tricarboxylic acid (TCA) cycle in sake. All metabolites are shown as μmol/L (n = 2). D) The relative values of pyridoxamine and pyridoxine (n = 2). Data are shown as means ± SEM.

The metabolites of the central metabolic pathway in each *ginjo* sake sample are shown in Fig 4. The content patterns of glucose and glucose 6-phosphate, which occur during the earlier stage of the glycolytic pathway, were similar (Fig 4B), whereas compounds at the later stage, including glycerol 3-phosphate, pyruvic acid, and lactic acid varied among sake samples. The level of pyruvic acid was extremely low in off-flavored sake, except for sample B4 and C5. Pyruvic acid is the last compound of the glycolytic pathway as well as an intermediate of alcoholic fermentation in *S*. *cerevisiae* (Fig 4A). Notably, the concentration of pyruvic acid in *ginjo* sake is strongly correlated with sake flavor and taste quality (Table 2). An extreme diminishing of pyruvic acid was found in some off-flavored sake (Fig 4B), suggesting that part of the generation of a specific off-flavor is correlated to pyruvic acid loss, and that this can be attributed to marked deterioration in yeast activity.

As depicted in Fig 4A, lactic acid is generally added to the modern yeast starter of *ginjo* sake (*shubo*). The lactic acid levels were almost the same in eleven sake samples; however, the concentration was clearly higher in the B6 and C2 sake samples.

Fig 4C shows the pattern of sake metabolites from the tricarboxylic acid (TCA) cycle. Levels of both *cis*-aconitic acid and isocitric acid, which are related to the TCA cycle, were higher in some off-flavored sake (Fig 4C). The content pattern of *cis*-aconitic acid and isocitric acid was quite similar in sake (Fig 4C). Notably, the content patterns of 2-oxoglutaric acid (α-ketoglutarate) and fumaric acid were completely different; a higher concentration of 2-oxoglutaric acid was found in high quality sake, and a higher concentration of fumaric acid was found in low quality sake (Fig 4C). Similarly, levels of pyridoxine and pyridoxamine, both of which are co-enzymes for amino transferase and are generally called vitamin B_6_, were also highly correlated with *ginjo* sake quality albeit in opposite directions (Fig 4D).

### Correlations of Metabolites with a Low Quality of Sake

**Fig 5:**
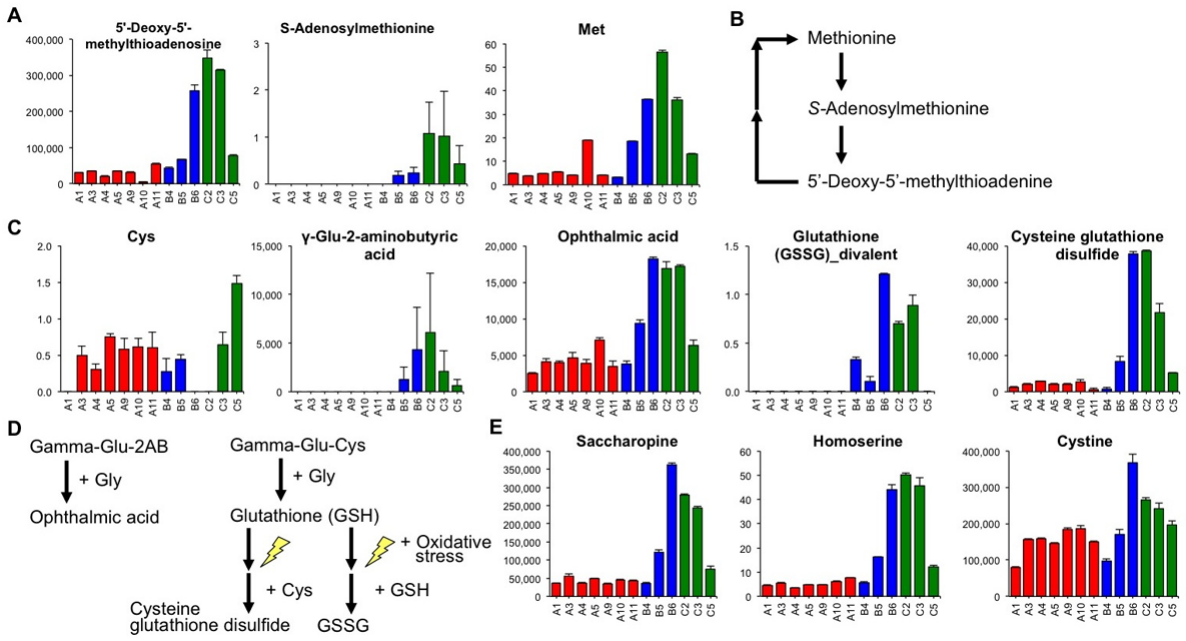
The patterns of representative metabolites associated with sake quality. A) Compounds involved in the methionine cycle. 5′-deoxi-5′-methylthioadenosine is shown as relative value. *S*-Adenosylmethionine and methionine are shown as μmol/L. B) A simple summarization of the methionine cycle pathway. C) Compounds involved in glutathione or ophthalmic acid biosynthesis pathways. Cysteine and glutathione (GSSG; oxiglutathione) are shown as μmol/L. Gamma-glutamyl-2-aminobutyric acid, ophthalmic acid, and cysteine glutathione disulfide are shown as relative values. D) The metabolites that participate in part of the glutathione (GSH) and ophthalmic acid biosynthesis pathways. E) Other amino acid-related chemical compounds associated with lower sake quality. Saccharopine and cystine are shown as relative values. Homoserine is shown as μmol/L. Data are shown as means ± SEM (n = 2).

Representative metabolites that were found at a higher amount in low quality sake and their relevant compounds are shown in Fig 5. Levels of methionine, *S*-adenosylmethionine, and 5′-deoxy-5′-methylthioadenine, which are some typical compounds in the methionine salvage cycle, were elevated in low quality sake (Fig 5A and 5B). Therefore, compounds in the methionine salvage cycle are expected to be involved in *ginjo* sake quality.

Compounds related to oxidized glutathione (i.e. GSSG, cysteine glutathione disulfide, γ-Glu-aminobutyric acid, and ophthalmic acid) but not to glutathione (GSH) were higher in some low quality sake (Fig 5C). Cysteine, a substrate for cysteine glutathione disulfide, could not be detected in B6 and C2 sake samples, which contained higher amounts of cysteine glutathione disulfide (Fig 5C), suggesting that cysteine was consumed to produce γ-Glu-Cys and cysteine glutathione disulfide (Fig 5D).

Other amino acid-related chemical compounds associated with low sake quality are shown in Fig 5E. The level of saccharopine, which consists of compounds derived from lysine and 2-oxoglutaric acid, and which was previously found in red wine [53], was higher in low quality sake (Fig 5E). The level of 2-oxoglutaric acid was lower in low-quality sake samples B6, C2, and C3, suggesting that a portion of 2-oxoglutaric acid could be utilized for saccharopine biosynthesis. The profile of homoserine was similar to that of saccharopine (Fig 5E). Cystine, the disulfide bonded form of cysteine, was slightly higher in low quality sake (Fig 5E).

### Correlations of Metabolites with Sake Off-Flavor

**Fig 6:**
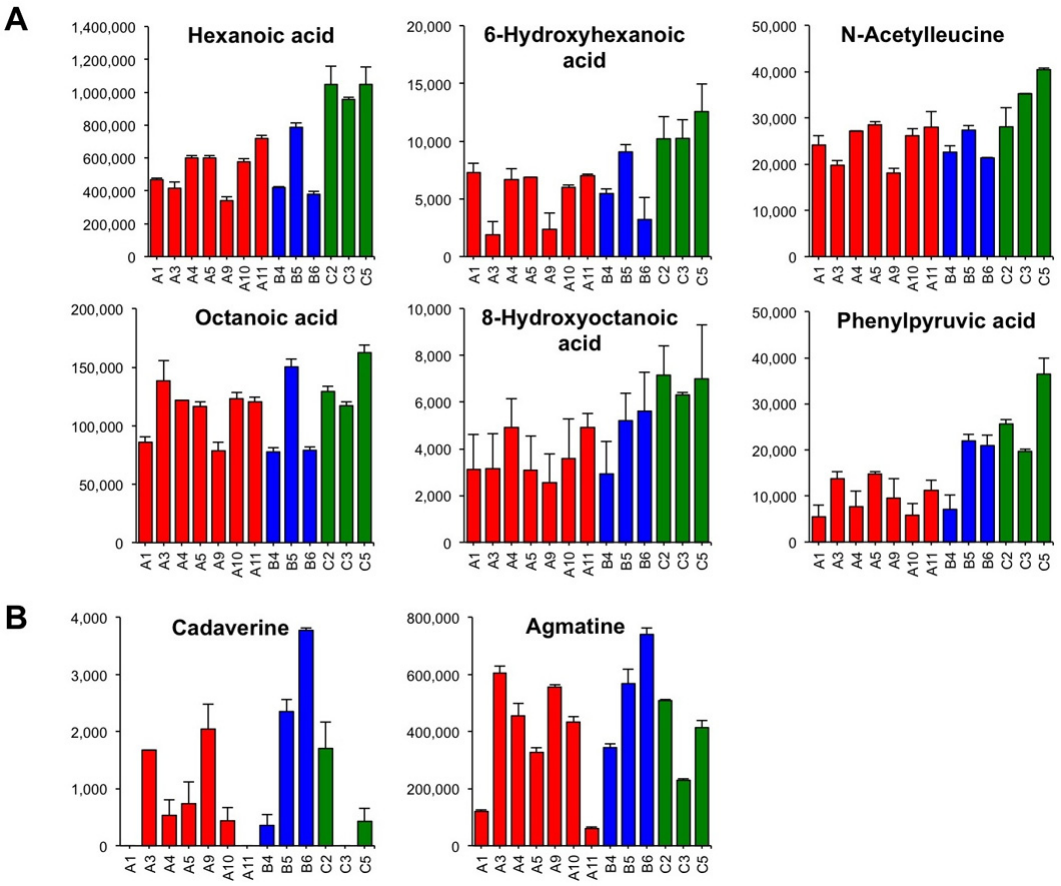
Chemical compounds contributing to fatty acid odor and inharmonious bitter taste. A) Representative chemical compounds involved in fatty acid odor generation are medium-chain fatty acids and medium-chain fatty acid analogues. B) Two representative polyamines (cadaverine and agmatine) involved in inharmonious bitter taste generation. All chemical compounds are shown as relative values. Data are shown as means ± SEM (n = 2).

We have reported that the CATA method of sensory evaluation can be applied to provide insight into the correlation analysis between the chemical compounds in sake and its organoleptic properties including off-flavor [6, 21]. Therefore, a correlation analysis was performed between sake metabolite levels and off-flavor (fatty acid odor and inharmonious bitter taste) according to the scores shown in Table 1. Compounds that were positively correlated with fatty acid odor included medium-chain fatty acids related compounds such as hexanoic acid, *N*-acetylleucine, 6-hydroxyhexanoic acid, 8-hydroxyoctanoic acid, and phenylpyruvic acid, as well as compounds associated with the methionine salvage cycle such as *S*-adenosylmethionine, methionine, and 5′-deoxy-5′-methylthioadenine (Table 3). Consistent with a previous report using GC [6], hexanoic acid was highly correlated with a fatty acid odor of *ginjo* sake. The content pattern of these compounds is shown in Fig 6A. Compounds that were negatively correlated with fatty acid odor were mevalolactone, quinic acid, 2-oxoglutaric acid, *N*,*N*-dimethylhistidine, and pyruvic acid (Table 3).

**Table 3:** Chemical compounds whose levels were positively or negatively correlated with the off-flavors of either fatty acid odor or inharmonious bitter taste.

Fatty acid odor		Inharmonious bitter taste	
Chemical compounds	*R*	Chemical compounds	*R*
Hexanoic acid	0.791	XA0012 (Unknown) ^(a)^	0.617
*N*-Acetylleucine	0.684	Cadaverine	0.594
*S*-Adenosylmethionine	0.655	*N*^2^-Succinylornithine	0.570
6-Hydroxyhexanoic acid	0.653	Penicillamine	0.565
Met	0.576	Histamine	0.559
8-Hydroxyoctanoic acid	0.572	Agmatine	0.549
Thr-Asp	0.571	N-Methylproline	0.546
1-Aminocyclopropane-1-carboxylic acid	0.569	Imidazole-4-acetic acid	0.544
Lys	0.566	*N*,*N*-Dimethylhistidine	0.517
5'-Deoxy-5'-methylthioadenosine	0.551	Methionine sulfoxide	0.515
Asp	0.543	Citric acid	0.515
Trp	0.541	*N*-Acetylalanine	0.511
Phenylpyruvic acid	0.538	Pipecolic acid	0.491
His-Glu	0.536	*N*^6^-Acetyllysine	0.489
Betaine aldehyde_+H2O	0.534	*N*-Methylputrescine	0.487
*N*-Acetylornithine	0.524	2-Isopropylmalic acid	0.471
Glucose 6-phosphate	0.522	Hypoxanthine	0.465
Homoserine	0.509	Succinic acid	0.447
Ala-Ala	0.504	4-Guanidinobutyric acid	0.428
Gln	0.495	Trimethylamine N-oxide	0.426
Benzocaine	0.474	β-Ala	0.417
Urocanic acid	0.472	*N*-Acetylmuramic acid	0.416
Ser	0.472	*O*-Acetylhomoserine	0.413
*N*-Acetylglucosamine 1-phosphate	0.465	4-(β-Acetylaminoethyl)imidazole	0.413
Ophthalmic acid	0.456	7-Methylguanine	0.404
Pyridoxamine	0.454	Putrescine	0.399
Octanoic acid	0.382		
3-Hydroxybutyric acid	-0.404		
2-Hydroxyvaleric acid	-0.428		
Ethanolamine phosphate	-0.432		
*N*-Methylproline	-0.453		
Cytidine	-0.465		
Pyridoxine	-0.474	Hexanoic acid	-0.359
Pyruvic acid	-0.481	Benzocaine	-0.362
*N*,*N*-Dimethylhistidine	-0.481	XC0040 (Unknown) ^(b)^	-0.363
2-Oxoglutaric acid	-0.481	Phosphorylcholine	-0.412
Quinic acid	-0.493	Arg	-0.481
Mevalolactone	-0.496	2'-Deoxycytidine	-0.635

^(a)^ The *m/z* is 166.0154 by anion detection mode.

^(b)^ The *m/z* is 174.0855 by cation detection mode.

Compounds that were positively correlated with inharmonious bitter taste included many polyamines such as cadaverine, agmatine, *N*-methylputrescine, and putrescine, as well as monoamines such as penicillamine and histamine, whereas Arg was negatively correlated (Table 3). In addition, several nucleic acid derivatives weakly correlated with inharmonious bitter taste. This result suggests that polyamines and amine-related compounds might be involved in an inharmonious bitter taste of *ginjo* sake.

In addition to fatty acid odor and inharmonious bitter taste, we performed correlation analysis between sake metabolites and “*koji-*like odor.” Citric acid, *cis*-aconitic acid, and isocitric acid, which are metabolites in TCA cycle, were positively correlated to “*koji-*like odor” (data not shown). Some *Aspergillus* species [54, 55] and sporulating *Bacillus subtilis* [56, 57] are known as being strong citric acid producers, suggesting that those tricarboxilic acids are also biomarkers for *A*. *oryzae* and might be increased by an enhancement of conidiation at the last stage of the rice mold making process.

### Protein Diversity in *Ginjo* Sake and Its Correlation with Metabolites

**Fig 7:**
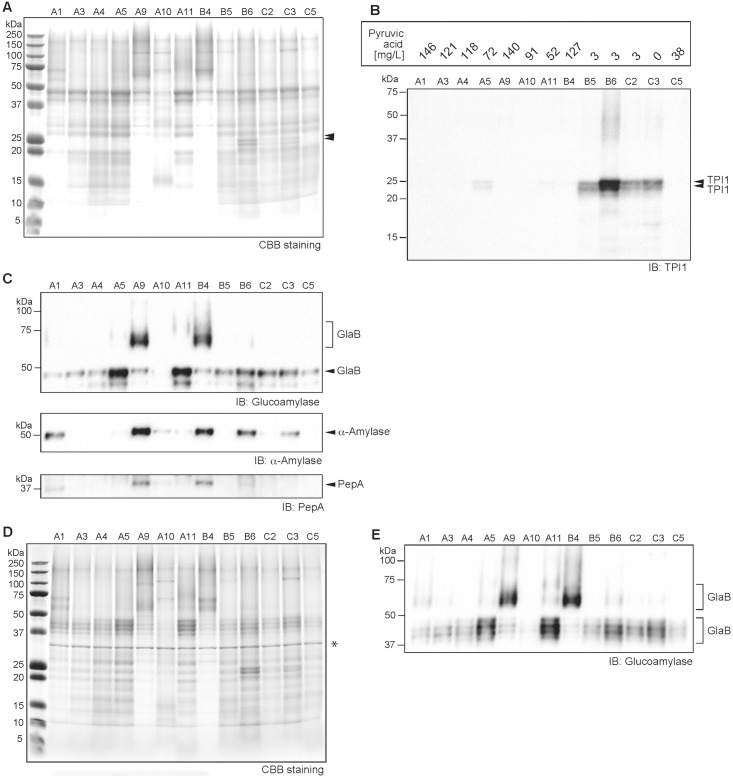
Protein profiles of 13 sake samples showing that yeast cellular protein leakage is linked to low sake quality. A) Overall protein profiling by Coomassie brilliant blue (CBB) staining after SDS-PAGE. For each sample, 10 μL of denatured protein was applied to a lane, and 5 μL of marker was utilized. Protein bands indicated by double black triangles are *Saccharomyces cerevisiae* triosephosphate isomerase (TPI). B) Immunoblot analysis for *S*. *cerevisiae* TPI1 in sake. Pyruvic acid concentrations in sake (n = 2) determined by colorimetric assay is shown in the upper rectangle. C) *Upper panel*; Immunoblot analysis for glucoamylase (GlaB) derived from *Aspergillus oryzae* in sake. The *N*-glycosylated forms of GlaB were detected at 47 kDa and as a blurred band beyond 65 kDa. *Middle panel*; Immunoblot analysis for α-amylase. *Lower panel*; Immunoblot analysis for the acid protease, PepA. D) Protein profiling following PNGaseF treatment by CBB staining after SDS-PAGE. For each sample, 10 μL of denatured protein was applied to a lane, and 5 μL of marker was applied. The asterisk at 34 kDa shows PNGaseF. E) Immunoblot analysis for glucoamylase (GlaB) following PNGaseF treatment of sake. The blurred band and multiple bands at 55–70 kDa and 37–50 kDa correspond to GlaB.

Metabolome analysis using CE–TOFMS showed that some sake (as represented by sample B6) contain relatively higher amounts of metabolites in addition to demonstrating the existence of a variety of metabolites among sake (Figs 2 and 3). Therefore, in order to investigate potential relationships between proteins and metabolites in sake, the residual proteins in sake were precipitated by acetone and characterized according to their polypeptide composition. As shown in Fig 7A, proteins in sake were found to exhibit diversity as seen for the metabolites, with some resemblances among sake. It was difficult to identify an overall apparent correlation between metabolites and protein profiles. Nevertheless, distinct protein bands located at 23 and 25 kDa were found in sake samples B5, B6, C2, and C3 (Fig 7A). The intensity of these two bands was strongest in sake sample B6, which is consistent with its metabolite level. To identify these two protein bands, MALDI-TOFMS analysis was performed. Both of these proteins were clearly identified as *S*. *cerevisiae* triosephosphate isomerase (TPI), which catalyzes the isomerization of a dihydroxyacetone phosphate to D-glyceraldehyde-3-phosphate in the glycolytic pathway. For further confirmation, we performed an immunoblot assay using a yeast TPI1-specific antibody as shown in Fig 7B. The results clearly indicated that the two bands located at 23 and 25 kDa for sake samples B5, B6, C2, and C3 were TPI polypeptides. The TPI detection level was strongly correlated with yeast debris-like odor and sulfur-like odor indices (Table 1). The pyruvic acid concentration in the sake samples was negatively correlated with TPI detection level (Fig 7B). Both dihydroxyacetone phosphate and D-glyceraldehyde-3-phosphate were not detected in the metabolome analysis. However, these results still clearly suggest that the leakage of one or more yeast intracellular proteins is associated with the abundance of metabolites in sake, which are also associated with lower quality of the final product.

The presence of major enzymes was analyzed by immunoblotting as shown in Fig 7C. As expected, we detected α-amylase and glucoamylase, both of which are produced by the rice mold *A*. *oryzae* in sake; however, unexpectedly, their contents exhibited large differences among sake samples (Fig 7C). Two major bands, a clear band at 47 kDa and a blurred band at 65–80 kDa, were detected for glucoamylase. To identify the protein band at 47 kDa and the blurred band at 65–80 kDa, MALDI-TOFMS analysis was performed. Both protein bands were identified as *A*. *oryzae* GlaB, and not GlaA. The GlaB is composed of a 493 amino-acid polypeptide [49], and its modification with *N*-linked glycosylation has been experimentally confirmed (Fig 7A, 7C, 7D and 7E). The α-amylase detection level was similar to that of the blurred 65–80 kDa band of GlaB (Fig 7C). The level of glucoamylase digestible dextrin and oligosaccharides in sake quantified by the colorimetric method indicated that the dextrin and oligosaccharide components were not associated with the residual GlaB and α-amylase levels in sake (data not shown). Acid protease (PepA) is a major endo-type protease that is also produced by *A*. *oryzae* during the middle stage of the rice mold-making process [58]. PepA levels were weakly correlated with the blurred 65–80 kDa band of GlaB and α-amylase in sake (Fig 7C), and the signal pattern was similar to that of α-amylase.

## Discussion

The relationship between the chemical compounds in sake and its organoleptic properties has been demonstrated recently utilizing the newly evolved metabolomic instrumentation and technologies. Previously, we had focused on premium *ginjo* sake and analyzed the volatile chemical compounds using a GC system. Because we were able to successively apply a simple method for sensory evaluation, “CATA,” to this analysis [6, 21], we performed a correlation analysis to evaluate the relationship of polar metabolites in *ginjo* sake determined using CE–TOFMS with both overall quality determined using QDA and off-flavors determined using the CATA method. Furthermore, to facilitate an understanding of comprehensive molecular relationships, protein analysis was integrated with polar metabolite analysis. Our results provided important observations regarding the processes of *ginjo* sake brewing.

Table 2 and Fig 4B show the correlation between the metabolites in the central metabolic pathway in *ginjo* sake and its organoleptic properties. In the Japanese sake brewery industry, a higher amount of pyruvic acid in the final stage of mash has been thought to lead to increased risk of generation of diacetyl off-flavors in bottled sake. However, in this study, as shown in Table 2, Figs 4B and 7B, pyruvic acid was barely detected in some off-flavor *ginjo* sake, suggesting that pyruvic acid loss is associated with the generation of off-flavors in *ginjo* sake. To confirm the relationship, we performed pyruvic acid quantification using another set of 64 *ginjo* sake samples (S1 Fig), and analyzed the correlation using sensory quality scores. The correlation coefficients between pyruvic acid and sensory quality scores (flavor quality, taste quality, fatty acid odor, yeast debris-like odor, and sulfur-like odor) were −0.26, −0.38, −0.44, −0.45, and −0.28, respectively (data not shown), thus verifying the relationship between pyruvic acid loss and the generation of off-flavors in *ginjo* sake. Although this conclusion appears contradictory, several reports have presented data supporting this supposition. For example, Ito et al. reported that the concentration of pyruvic acid in the final stage of sake mash fermented using cerulenin-resistance *ginjo* sake yeast tended to sharply decrease to almost 0 mg/L [59]. In addition, Iwano et al. reported that the concentration of pyruvic acid was only slightly, i.e., not significantly, correlated with *ginjo* sake quality (*R* = −0.188) [25]. Together, these results suggest that for *ginjo* sake, pyruvate loss rather than maintenance in the last stage of sake mash could be the critical factor for generation of off-flavors. Therefore, for *ginjo* sake brewing, it may be better to stop sake brewing and squeeze within a few days when the pyruvic acid level decreases to 0 mg/L in the final stage of the sake mash.

Unique molecular content patterns were observed for fumaric and 2-oxoglutaric acids, both of which are key metabolites in the TCA cycle (Table 2 and Fig 4C). 2-Oxoglutaric acid is known as a substrate for inorganic nitrogen assimilation for the conversion to glutamate *via* glutamate dehydrogenase. In turn, glutamate can be a substrate for aminotransferase with other amino acids to produce 2-oxoglutaric acid. Using the laboratory strain of *S*. *cerevisiae* BY4742 under aerated condition, Nakayama et al. showed that the turnover speed of 2-oxoglutaric acid was the slowest for TCA cycle compounds, whereas that of fumaric acid was the fastest [60], indicating that the flux behavior of the two compounds differs considerably. In anaerobic fermentation, such as in *ginjo* sake brewing conditions, fumaric acid can be reduced by fumarate reductase to produce succinic acid [61]; however, marked succinic acid variation was not observed in our study (Fig 4C). Overall, the patterns of 2-oxoglutaric acid, fumaric acid, and succinic acid are enigmatic and the research to elucidate their relevance to a physiological state is ongoing. Recently, it has been reported that 2-oxoglutaric acid is associated with extended lifespan in *Caenorhabditis elegans* [62] while fumaric acid is associated with shortened lifespan in mouse kidney cells [63]. Important roles for 2-oxoglutaric acid and the ratio of 2-oxoglutaric acid/succinic acid in mammalian embryonic stem cells have been also reported [64]. While the roles of these major metabolites in microorganisms including *S*. *cerevisiae* have not been reported, clues might be provided through analysis of whether their levels are reflected by physiological state of the organism. Further research targeted to these compounds is required to address these issues.

Oxidized glutathione related compounds (i.e. GSSG, cysteine glutathione disulfide, γ-Glu-aminobutyric acid, and ophthalmic acid) but not GSH were higher in some low quality sake (Fig 5C). In *S*. *cerevisiae*, γ-Glu-Cys is synthesized by Glu and Cys *via* GSH1 [65]. GSH1 can also catalyze the ligation of Glu and 2-aminobutyric acid (2AB) to produce γ-Glu-2AB because its homologue in *E*. *coli* and mammals, γ-glutamylcysteine synthetase, catalyzes the ligation of Glu and 2AB [66]. Subsequently, γ-Glu-Cys and γ-Glu-2AB are conjugated with Gly via yeast GSH2 to produce GSH and ophthalmic acid, respectively (Fig 5D) [67, 68]. Ophthalmic acid is known as a compound produced when cells are exposed to oxidative stress that causes a deficiency in cysteine molecules [68]. When cells are in a reducing state, GSH is produced, whereas when cells are in an oxidative state, compounds related to oxidized glutathione relatives (i.e., GSSG, cysteine glutathione disulfide, γ-Glu-aminobutyric acid, and ophthalmic acid) would be produced. Therefore, oxidative stress in microorganisms during the sake brewing process might be associated with *ginjo* sake quality.

The correlation of many amino acids with low quality sake has been well known in the field of sake brewing industry, and has been recently supported by metabolomics approaches [3, 5, 25]. Consistent with previous reports, in this study, we found that many amino acids and dimethylarginine were correlated with low quality *ginjo* sake, whereas arginine and some types of dipeptides and tripeptides were correlated with high quality *ginjo* sake (Table 2). We previously reported that many varieties and large amounts of dipeptides are contained in sake compared to wine and beer by LC–MS analysis [53]. In addition, several cyclic-dipeptides have been correlated with sake quality and a pleasant flavor, ethyl caproate, through GC × GC–TOFMS analysis [21]. These results suggest that some types of dipeptides might be involved in *ginjo* sake quality. Therefore, future studies should address whether the amount and types of dipeptides in sake are associated with its quality.

In previous studies, we analyzed the volatile compounds of the same sake samples used in this study using a GC system [6, 21], but we could not distinguish the metabolite content pattern of sake sample B6. In this study, comprehensive polar metabolite analysis by CE–TOFMS was able to reveal that sake sample B6 contained relatively higher amount of metabolites as well as an enhanced variety of metabolites among sake samples (Figs 2 and 3). On the other hand, protein analysis clearly showed that sake sample B6 contained notable protein bands at 23 and 25 kDa (Fig 7A and 7B), which were found to be derived from *S*. *cerevisiae* TPI. Detection of intracellular TPI in sake by the protein leakage indicates a rise in the ratio of yeast cell death at the last stage of sake mash causing degradation of cell membrane (cell wall). In support of this mechanism, the remarkable reduction of pyruvic acid concentration in some *ginjo* sake was associated with an increased TPI level (Fig 7B and S1 Fig). In addition to protein leakage from yeast, the difference of mechanical compression at the last step of sake brewing process, *Joso*, by each brewery could have also contributed to metabolite and protein level diversity among sake samples. The reason why TPI was the only remarkable protein in lower-quality sake is unknown.

In this study, we observed large differences in GlaB profiles among sake samples (Fig 7C and 7E), although these differences did not seem to affect *ginjo* sake quality. We also confirmed that a single 65 kDa GlaB protein was expressed during the solid-state rice mold making process (data not shown). The 47 kDa GlaB may be a partially digested form of the 65 kDa GlaB, whereas the blurred 65–80 kDa GlaB could have resulted from differences in glucoamylase genes, which could produce alternative splicing variants of GlaB or enzyme resistance. The differences in *glaB* gene expression could have resulted from the *A*. *oryzae* species used in the rice mold making process. Because it is a general practice of *A*. *oryzae* suppliers to mix several *A*. *oryzae* species during the formulation of rice mold seeds to be reach a desirable enzymatic activity, proteins from a variety of *A*. *oryzae* species are likely contained in sake. GlaA is 65 kDa polypeptide of *N*-linked glycosylated secreted protein that is only expressed in submerged culture [69, 70]. However, in MALDI-TOFMS analysis, GlaA was not detected, which suggests that GlaA was not expressed to a detectable level in sake mash after the solid-state rice mold making process.

## Conclusions

In conclusion, the results of this study clearly demonstrate that certain polar metabolites in *ginjo* sake strongly correlate with its quality, and others correlate with its off-flavor. However, questions remain regarding the reasons or mechanisms by which these chemicals or their molecular rations might be associated with sake quality and off-flavor. To address this, further investigation of targeted chemicals and molecular biological approaches using cell culture systems will be required. In addition, this study identified that several low quality sake samples contained higher amounts of polar metabolites that corresponded to a particular protein, TPI, in *ginjo* sake. The integrated approach combining successive metabolomics and protein analysis in sake presented here was the first demonstration of its application in the field of sake brewing, suggesting that it might possibly be applicable to other food or beverage fields as well.

## Supporting Information

S1 Fig Relationship between yeast TPI protein level and pyruvic acid concentration in 64 *ginjo* sake samples. TPI was detected by immunoblot analysis in sake samples that were entered in the Annual NRIB National New Sake Awards competition held in 2009 (09), 2010 (10), and 2011 (11). Highly ranked sake samples are described as ‘A’, inharmonious bitter-tasting sake samples are described as ‘B’, and sake samples with a fatty acid odor are described as ‘C’. Pyruvic acid concentration in sake determined by colorimetric assay is shown in graphs. As part of the sensory evaluation score, yeast debris-like odor and sulfur-like odor indices are also displayed.
(TIFF)

## Abbreviations

2-AB: 2-aminobutyric acid
CATA: check-all-that-apply
CBB: Coomassie brilliant blue
CE–TOFMS: capillary electrophoresis time-of-flight mass spectrometry
ESI–MS: Electrospray ionization-mass spectrometry
GC–MS: gas chromatography coupled with mass spectrometry
MALDI: matrix-assisted laser desorption-ionization
GSH: glutathione
GSSG: oxiglutathione
HMT: Human Metabolome Technologies
KLH: keyhole limpet hemocyanin
PCA: Principal component analysis
PepA: Acid protease
QDA: quantitative descriptive analysis
TCA: tricarboxylic acid
TPI1: triosephosphate isomerase 1
