# Supplementary figures and images for: Different Polar Metabolites and Protein Profiles between High- and Low-Quality Japanese Ginjo Sake
Source: PLoS One. 2016 Mar 3;11(3):e0150524. doi: 10.1371/journal.pone.0150524 (PMC4777507; doi:10.1371/journal.pone.0150524)

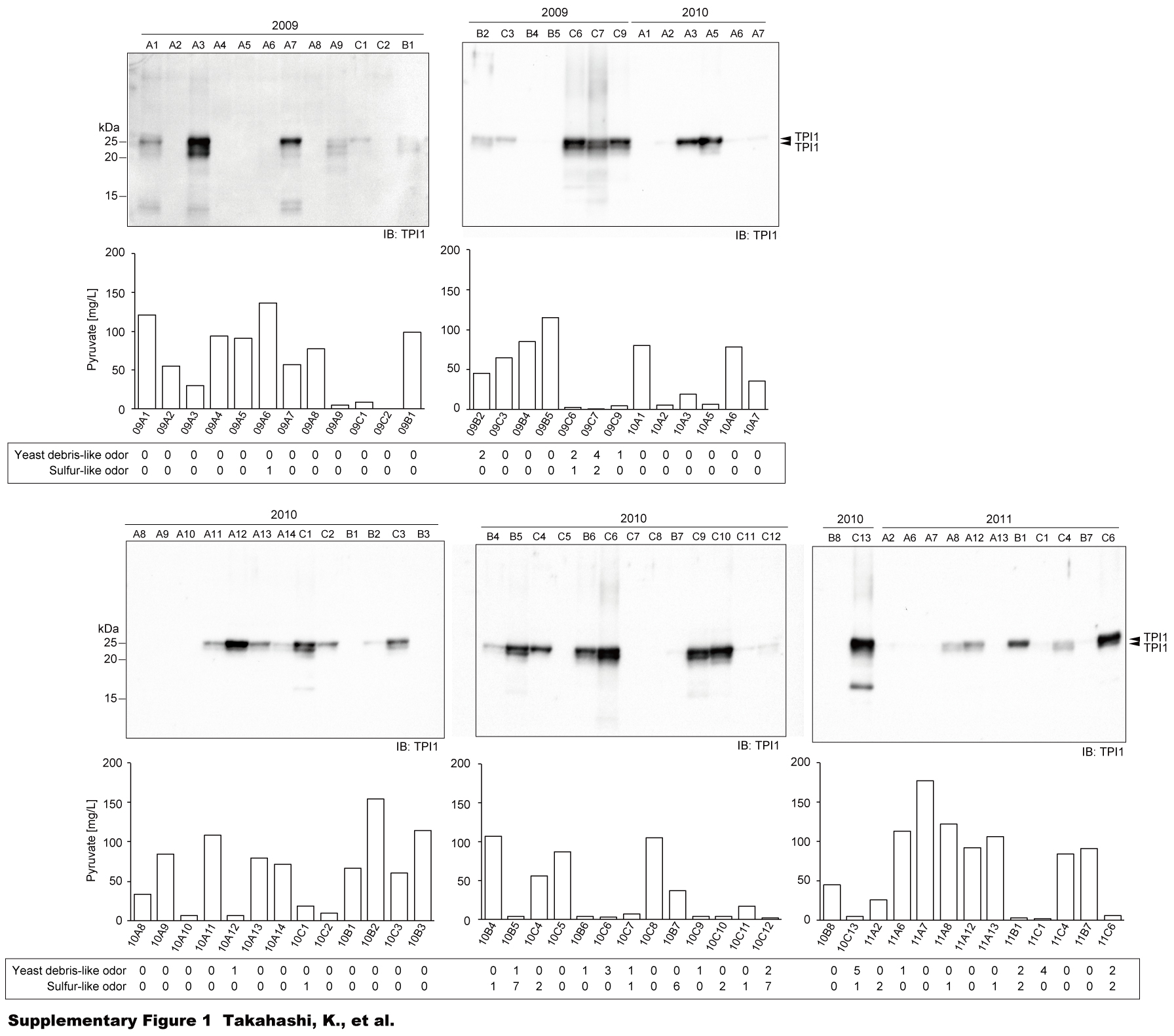

Supplement: S1 Fig — TPI was detected by immunoblot analysis in sake samples that were entered in the Annual NRIB National New Sake Awards competition held in 2009 (09), 2010 (10), and 2011 (11). Highly ranked sake samples are described as ‘A’, inharmonious bitter-tasting sake samples are described as ‘B’, and sake samples with a fatty acid odor are described as ‘C’. Pyruvic acid concentration in sake determined by colorimetric assay is shown in graphs. As part of the sensory evaluation score, yeast debris-like odor and sulfur-like odor indices are also displayed. (TIFF) [file pone.0150524.s001.tiff]
